# Time Course of the Cross-Over Effect of Fatigue on the Contralateral Muscle after Unilateral Exercise

**DOI:** 10.1371/journal.pone.0064910

**Published:** 2013-05-31

**Authors:** Aude-Clémence M. Doix, Fabrice Lefèvre, Serge S. Colson

**Affiliations:** 1 University of Nice Sophia-Antipolis, Nice and University of Toulon, Laboratory of Human Motricity, Education and Health, Nice, France; 2 Norwegian University of Science and Technology, Department of Human Movement Science, Trondheim, Norway; 3 International Health & Performance Institute, Barcelona, Spain; The University of Queensland, Australia

## Abstract

We investigated the cross-over effect of muscle fatigue and its time course on the non-exercising contralateral limb (NEL) after unilateral fatiguing contractions of the ipsilateral exercising limb (EL). For this purpose, 15 males performed two bouts of 100-second maximal isometric knee extensions with the exercising limb, and neuromuscular function of both the EL and NEL was assessed before (PRE), after a first fatiguing exercise (MID) and after a second fatiguing exercise (POST). Maximal voluntary isometric torque production declined in the EL after the first bout of exercise (−9.6%; *p*<0.001) while in the NEL, the decrease occurred after the second bout of exercise (−10.6%; *p*<0.001). At MID, torque decline of the EL was strictly associated to an alteration of the mechanical twitch properties evoked by neurostimulation of the femoral nerve (*i.e.*, peak twitch torque, maximal rate of twitch development). According to these markers, we suggest that peripheral fatigue occurred. At POST, after the second bout of exercise, the voluntary activation level of the knee extensor muscles was altered from PRE (−9.1%; *p*<0.001), indicating an overall central failure in both the EL and NEL. These findings indicate that two bouts of unilateral fatiguing exercise were needed to induce a cross-over effect of muscle fatigue on the non-exercising contralateral limb. Differential adjustments of the motor pathway (peripheral fatigue *vs*. central fatigue) might contribute to the respective torque decline in the EL and the NEL. Given that our unilateral fatiguing exercise induced immediate maximal torque reduction in the EL and postponed the loss of torque production in the NEL, it is also concluded that the time course of muscle fatigue differed between limbs.

## Introduction

Muscle fatigue is a broad research field and its mechanisms are widely investigated, but data are scant regarding the cross-over effect of fatigue. This cross-over effect relates to the fact that one type of activity can negatively impact other types of action. For example, Mosso [1] initially observed a decreased endurance in a manual task after a day of intense intellectual activity. Recently, Kennedy et al. [2] showed that the maximal voluntary contraction of the plantar flexor muscles was decreased after bilateral sustained handgrip contractions. However, the cross-over effect of fatigue has been mainly investigated after a unilateral exercise onto the performance of the contralateral homologous muscle [3]–[8] or limb [9]–[12].

Generally, muscle fatigue is defined as a progressive and transient reduction of maximal force production induced by sustained or repeated muscle contractions, no matter whether the task can be sustained or not [13], [14]. This definition implies that the maximal force production of the non-exercising contralateral muscle must decrease to conclude that cross-over effect of muscle fatigue occurred. So far, only two studies have shown that the maximal voluntary contraction (MVC) of the rested contralateral muscle declined after a unilateral fatiguing exercise of the ipsilateral homologous muscle [7], [8]. Martin and Rattey [7] reported an average reduction of 16% in women and 25% in men of the contralateral MVC of the knee extensor muscles after a 100-second sustained maximal isometric knee extension. In the first dorsal interosseous muscle of the contralateral hand, Post et al. [8] observed an average MVC decline of 10% in a group of men and women after two distinct fatiguing protocols consisting of either a 120-second sustained isometric MVC or a submaximal intermittent exercise at 30% of MVC maintained until exhaustion. Other authors have highlighted that the motor performance of the rested contralateral limb was negatively affected by using biomechanical [9] or postural control approaches [10], [11]. Conversely, it is noteworthy that many studies have failed to observe significant MVC reduction of the homologous contralateral muscles after a unilateral fatiguing exercise either for muscles of the upper limbs [3]–[5] or the lower limbs [6],[10], [11]. The heterogeneity of the experimental procedures can easily account for these discrepancies and not all the results can be equally compared. As a consequence, the cross-over effect of muscle fatigue remains to be debated.

The manifestations of muscle fatigue can occur through either peripheral or central pathways. Specifically, peripheral fatigue refers to a failure of the muscle in generating force at the level of or distal to the neuromuscular junction [15]. Central fatigue, instead, relates to a progressive reduction in voluntary activation that encompasses supraspinal and spinal circuitry. In the literature, most of aforementioned studies agreed that the cross-over effect of fatigue pertains to central fatigue mechanisms. Nonetheless, few have effectively investigated the cross-over effect of central fatigue [3], [5]–[8]. Indeed, two studies have found that the reduced MVC of the non-exercising contralateral muscle was associated to a significant decline in voluntary activation [7], [8]. As mentioned earlier, Post et al. [8] also examined the effect of sustained maximal and repetitive submaximal exercise, and their results indicated that the maximal effort induced a more pronounced decrease of voluntary activation in the non-exercising contralateral muscle. In line with this observation, Kennedy et al. [2] concluded that a maximal fatigue protocol of handgrip contractions affected voluntary activation and MVC of the ankle plantar flexor muscles more severely than a submaximal protocol. Overall these observations emphasised that the cross-over effect of fatigue may relate to the intensity of the contraction performed. Conversely, high-intensity single-leg cycling did not compromise maximum power capacity of the rested contralateral limb nor the maximum isometric handgrip force [12]. For all these reasons, the mechanisms underlying the cross-over effect of fatigue have yet to be clarified.

An interesting approach to delineate the effect of a unilateral fatiguing exercise on the maximal force production of the non-exercising contralateral muscle could be implemented by analysing the time course of the cross-over effect of fatigue. At present, there is evidence in the literature supporting this approach. Indeed, it has been suggested that multiple muscle contractions progressively impairs voluntary activation and thus increases the cross-over effect of fatigue [3], [5]. Therefore, the aim of the present study was to investigate the time course of muscle fatigue in both knee extensor muscles after unilateral maximal isometric fatiguing exercise consisting of two bouts of 100-second MVC. It was hypothesised that the first bout of exercise would induce muscle fatigue in the exercising limb, and we expected that the cross-over effect of fatigue of the non-exercising contralateral limb would occur after the second bout of exercise.

## Methods

### Participants

Fifteen healthy males (age: 21.7±3.1 years; height: 181±5 cm; body mass: 76.8±7.9 kg; mean ±SD) accepted to take part in the study. Participants were physical education students and were recreationally active (≈ 5 to 10 hours a week). Participants did not report any pathology, neurological complications, muscular, tendon or joint injury within six months prior to the study.

### Ethics Statement

Participants were informed about potential risks and gave informed written consent prior to enrolment in the investigation. The experiment was approved by the Ethics Committee on Human Experiments in Life and Health Sciences of the University of Nice - Sophia Antipolis and in accordance with the Helsinki Declaration (1964).

### Equipment Set-up

#### Torque measurements

A stationary Biodex® dynamometer (System 3 Pro; Biodex Medical Systems, Shirley, NY, USA) in isometric mode was used to record isometric MVCs and the evoked contractions of knee extension. The axis of the dynamometer was aligned with the anatomical axis of the knee flexion-extension, and the lever arm was attached on the shank, about 2 cm above the lateral malleolus of the ankle. Participants were positioned on the seat with a hip angle of 120° and a knee angle of 60° (0° being considered as the full knee extension). Upper-body movements of participants were constrained by two cross-over shoulder belts and a belt across the abdomen. Participants were allowed to grip cross-over shoulder belts during the testing procedure, but they were not allowed to hold the seat. To allow semitendinosus (ST) surface electromyography recordings, a board (thickness ≈5 cm) was placed underneath the subject with a hole where the electrodes were placed to avoid any compression between the surface electrodes and the wire on the seat.

#### Surface electromyography (sEMG) recordings

Bi-polar sEMG electrodes (silver chloride, recording diameter of 1cm, 2 cm inter-electrode distance, Contrôle Graphique Médical, Brie-Comte-Robert, France) were positioned over the VL (Vastus Lateralis), the VM (Vastus Medialis), the RF (Rectus Femoris) and the Semitendinosus (ST) on both limbs, according to the SENIAM recommendations [16]. The reference electrode was placed on the medial bony party of the right wrist. Low-resistance impedance between electrodes (<5 kΩ) was obtained abrading the skin and cleaning it with alcohol. A Biopac MP 100 system (Biopac systems, Inc., Holliston, MA, USA) was used to record sEMG data at a sampling rate of 2000 Hz. EMG signals were amplified with a bandwidth frequency ranging from 1 Hz to 500 Hz (common mode rejection ratio = 11 dB; impedance input = 1000 MΩ; gain = 1000).

#### Femoral nerve stimulation

Femoral nerve stimulation was induced using an electrical stimulator (Digitimer DS7AH, Digitimer, Herthforshire, United-Kingdom) to evoke the compound muscle action potential and associated mechanical response. The femoral nerve was stimulated with a tungsten cathode ball electrode (0.5 cm diameter) pressed onto the femoral triangle by the same experimenter during the whole session. The anode was a 45 cm^2^ rectangular electrode (Stimex, Wetzlar, Germany) placed between the great trochanter and the iliac spine. The stimulus was a 400 V and 2 ms rectangular pulse. The optimal intensity of stimulation was set by increasing intensity by 10 mA steps. The maximal intensity was reached once the compound muscle action potential (M_max_) and the torque output of the associated mechanical response were found to be maximal and stable. Then, to elicit a supramaximal stimulation, the maximal stimulation intensity was increased by 10%. This intensity was used to induce doublet stimuli, separated by 10 ms at 100 Hz. Individual supramaximal intensities were between 70 and 140 mA.

### Experimental Procedure

Participants were required to attend two sessions at the laboratory. The first session served to familiarise the participants with the equipment and testing procedures. At the second session, participants were equipped with sEMG electrodes on both lower limbs. The participants performed a 5-minute standardised warm-up at 2 watts.kg^−1^ on a cycling ergometer (Monark, 818E, Vansbro, Sweden) with a pedaling frequency of 70 rpm [17]. After warming-up, volunteers were transferred and secured to the isokinetic dynamometer. The neurostimulation intensity was rapidly adjusted. While fatiguing exercises were performed unilaterally, hereby the exercising limb (EL), neuromuscular tests were done on both the EL and the non-exercising limb (NEL) to evaluate fatigue on both limbs.

#### Pre-, mid- and post-fatigue neuromuscular tests

**Figure 1:**
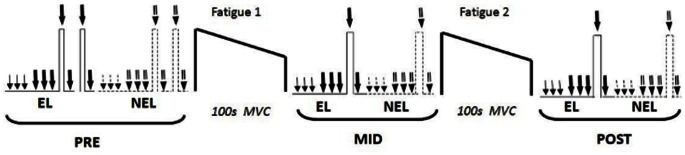
Graphical overview of the experimental protocol. Neuromuscular tests comprised single stimuli (single arrows), doublet stimuli (double arrows), MVC with superimposed doublet stimulus and followed by a doublet stimulus delivered at rest. Two MVCs of the exercising limb (EL; uninterrupted line) and the non-exercising limb (NEL; dashed lines) were performed at PRE, while only one was respectively realised at MID and POST. Testing order for the EL and NEL was randomly selected. The fatiguing exercise of the EL consisted of two bouts of 100-second MVC.

Figure 1 illustrates the experimental design and procedures used for data collection. A sequence of neuromuscular tests was performed before (PRE), after a first fatiguing exercise (MID) and after a second fatiguing exercise (POST). These tests were conducted as follows: 1) three single stimuli were delivered at rest, separated by 5 seconds; 2) three doublet stimuli spaced by 5 seconds delivered at rest; 3) two 4-second isometric MVCs of knee extension (separated by a 2-minute rest) with doublet stimuli delivered respectively over the isometric plateau (superimposed doublet), and 4 seconds after the MVC (potentiated doublet) [18]. Strong verbal encouragement was provided by the experimenters throughout MVCs. During PRE, two MVCs were achieved, while only one MVC was carried out during MID and POST tests assessments. Although the sequence of neuromuscular tests was standardised, the order of EL or NEL assessments was randomly selected for the PRE, MID and POST tests.

#### Fatiguing exercise

The fatiguing exercise consisted of two bouts (thereafter called Fatigue 1 and Fatigue 2) of 100-second MVC knee extension of the EL. The side of the EL (*i.e.*, right or left) was randomly selected across the participants. No visual feedback was provided to participants and they were asked to perform an all-out effort, and received strong verbal encouragement. Participants were also asked to relax the NEL during fatiguing tasks. To avoid teleoanticipation [19], [20], the participants were not aware that two bouts of MVC were to be performed.

### Data Analysis

#### Torque and sEMG recordings

The MVC was considered as the mean value over a 500-ms period when the torque output reached a maximal plateau. In PRE tests, the best MVC trial was analysed. All sEMG were analysed over the same window width. Root mean square (RMS) of the VL, VM and RF muscles were calculated (AcqKnowledge® 3.8.2, BiopacSystems, Inc., Holliston, MA, USA) and normalised to their respective M_max_ peak-to-peak amplitude (*i.e.*, RMS/M_max_ ratio) obtained at PRE, MID and POST. This normalisation procedure reduces the variability in the EMG signals due to changes at the skin level and therefore allows the interpretation of the RMS/M_max_ modification as a central nervous adaptation. RMS of the ST muscle was computed and the coactivation level was expressed by the ratio between the ST RMS value and the sum of the RMS/M_max_ values of VM, VL and RF muscles [21]. During the fatiguing exercises, torque production, torque-time integral, as well as sEMG values of the different muscles were analysed in ten consecutive periods that represented 10% of the total duration (100%) of each fatiguing bout. The RMS values of the VM, VL and RF were normalised to the M_max_ of the respective muscles obtained before each fatiguing exercise (PRE and MID). The coactivation level was expressed by the ratio between the ST RMS value and the sum of the RMS/M_max_ values of VM, VL and RF muscles computed during the fatiguing exercise.

#### Evoked responses

M_max_ signals elicited by the three single stimuli delivered at rest were averaged and peak-to-peak amplitude (A) and the peak-to-peak duration (D) of the VM, VL and RF muscles were analysed. Mechanical twitch responses were obtained (average of the three) from single-peak twitch (Pts). The mechanical response induced by doublet stimuli after the MVC was considered as the potentiated twitch (PtdPot). The maximal voluntary activation level of knee extensors was calculated using the following formula [22]:
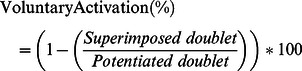



A correction was consistently applied to the original equation when the superimposed doublet was elicited slightly before or after the real MVC [23]. In these cases, the maximal voluntary activation level was calculated as follows:
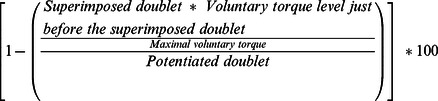



#### Statistical analysis

Statistical processing was performed in Statistica (Statsoft, version 8.0 Tulsa, OK, USA). The Kolmogorov-Smirnov test was used to test whether outcome measures were normally distributed. The statistical significance was set at *p*<0.05. Since data were normally distributed, a repeated measures two-way ANOVA (limb × time) was performed to assess fatigue-induced changes between pre-, mid- and post-fatigue neuromuscular tests. Separate two-factor ANOVA (fatiguing exercise × periods) with repeated measures on fatiguing exercise and periods were used to compare torque, RMS/M_max_ values and coactivation during the fatiguing exercises. Post-hoc analyses (Bonferroni) were used to test for differences among pairs of means when appropriate. A Pearson correlation coefficient was used to assess the relation between the MVC torque production and the voluntary activation level changes. Effect size was computed from partial eta^2^ values (η^2^p). Unless specified, all data are expressed as means±SE (standard error of the mean) in the entire manuscript and in the tables and figures.

## Results

Regardless of the variable, no differences between the EL and the NEL have been observed during the PRE test, before the first fatiguing exercise.

### MVCs, Voluntary Activation and sEMG

**Figure 2:**
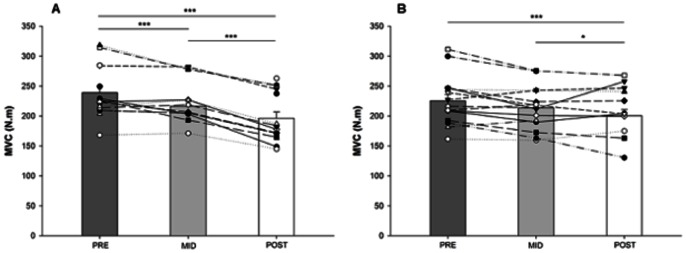
Maximal voluntary isometric torque. Maximal voluntary isometric torque tests of the knee extensor muscles measured at PRE, MID and POST tests for the exercising limb (A) and the non-exercising limb (B). Columns represent group mean values, while triangles, squares black and white symbols show individual values. Error bars are the standard error of the group mean. Significant differences *p*<0.05 (*) and *p*<0.001 (***).

A significant limb × time interaction was observed for MVCs values (F = 3.26; *p*<0.05; η^2^p = 0.10). In the EL (Figure 2A), a significant reduction in maximal torque was observed between PRE and MID tests (−9.63±1.67%; *p*<0.001), MID and POST tests (−9.24±1%; *p*<0.01), and between PRE and POST tests (−17.91±1.93%; *p*<0.001) whereas, the maximal torque decline of the NEL was only significant between PRE and POST test (−10.58±3.45%; *p*<0.001; Figure 2B).

**Figure 3:**
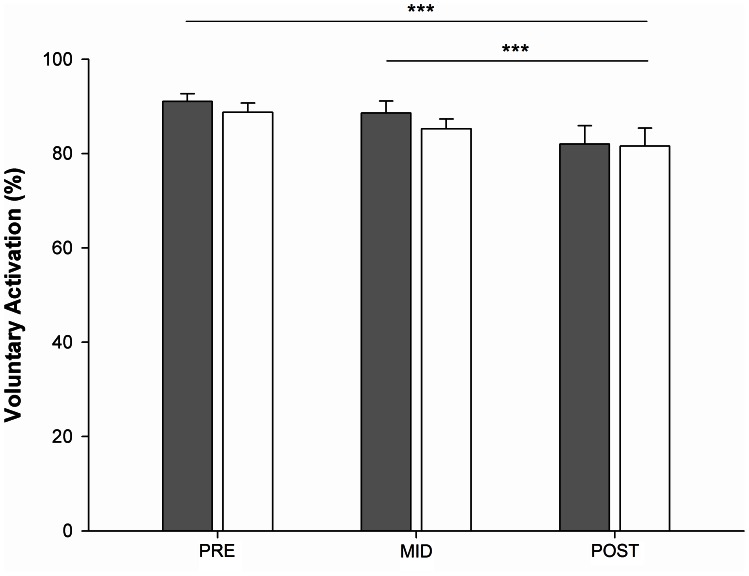
Voluntary activation level of the knee extensor muscles. Voluntary activation level measured at PRE, MID and POST tests for the exercising limb (in grey) and the non-exercising limb (in white). Columns represent group mean values and error bars are the standard error of the group mean. Significant differences *p*<0.001 (***).

No limb × time interaction was noted for voluntary activation of the knee extensor muscles, but a significant time effect was observed (F = 9.98; *p*<0.001; η^2^p = 0.26; Figure 3). Regardless of the tested limb, voluntary activation was significantly depressed from MID and POST tests (−6.07±2.22%; *p*<0.05), as well as from PRE to POST test (−9.14±2.74%; *p*<0.001).

A significant correlation (r = 0.451; p<0.05) was found between the relative decline in MVC torque and the relative loss in voluntary activation of both limbs pooled data. This relationship indicated that the greater the MVC loss was, the greater the voluntary activation reduction.

No significant change was found in the RMS/M_max_ ratios of VL, RF, and VM muscles, nor in the coactivation level of the ST muscle recorded during MVCs (*p*>0.05; Table 1).

**Table 1:** sEMG RMS/M_max_ values of the knee extensors and coactivation level for both limbs.

		PRE				MID				POST			
		RMS/MVL	RMS/M RF	RMS/M VM	CO-A	RMS/M VL	RMS/M R	RMS/M VM	CO-A	RMS/M VL	RMS/M RF	RMS/M VM	CO-
**EL**	**Mean** **(SE)**	0.065(0.008)	0.111(0.015)	0.068(0.009)	0.307(0.081)	0.066(0.007)	0.106(0.012)	0.077(0.013)	0.217(0.026)	0.062(0.007)	0.095(0.010)	0.067(0.010)	0.213(0.024)
**NEL**	**Mean** **(SE)**	0.077(0.013)	0.093(0.011)	0.067(0.010)	0.178(0.019)	0.077(0.012)	0.088(0.008)	0.064(0.009)	0.181(0.016)	0.078(0.012)	0.081(0.008)	0.066(0.012)	0.186(0.022)

Mean and standard error of sEMG RMS/M_max_ values of the vastus lateralis (VL), vastus medialis (VM), rectus femoris (RF) muscles and coactivation level (CO-A)of the semitendinosus (ST) muscle obtained during PRE, MID and POST tests for both the exercising (EL) and non-exercising (NEL) limbs. VL, VM and RF values were normalised to the respective muscle M_max_ responses of the test.

### Mechanical Twitch and M_max_ Responses

A significant limb × time interaction was found (F = 3.37; *p*<0.05; η^2^p = 0.11) for the mechanical twitch response evoked by single stimulus (Pts). A significant decrease was observed in the EL between PRE and POST tests (−24.39±7.21%; *p*<0.001), while no modification was found in the NEL (Table 2).

**Table 2:** Mechanical responses evoked by single stimulation and potentiated doublet for both limbs.

		Pts (N.m)			PtdPot (N.m)		
		PRE	MID	POST	PRE	MID	POST
**EL**	**Mean (SE)**	37.97 (2.68)	32.61 (3.17)	28.66*** (3.56)	102.73 (5.22)	97.09 (5.36)	89.19## (5.16)
**NEL**	**Mean (SE)**	35.92 (2.95)	34.33 (3.74)	34.45 (3.75)	94.40 (5.34)	94.24 (5.55)	91.11## (5.83)

Mean and standard error of mechanical twitch response evoked by single stimulation (Pts), potentiated twitch evoked with a double stimulation (PtdPot) measured at PRE, MID and POST tests for the exercising limb (EL) and the non-exercising limb (NEL). Significant differences of the EL values between PRE and POST: *p*<0.001 (***). Significant differences of pooled data for the exercising limb (EL) and the non-exercising limb (NEL) between PRE and POST: *p*<0.01(##).

A significant time effect was noted for potentiated doublet-peak twitch (PtdPot; F = 5.54; *p*<0.01; η^2^p = 0.17). Independently of the limb, PtdPot values decreased from PRE to POST test (−7.20±2.95%; *p*<0.01).

No significant interaction was observed for M_max_ responses of VM, VL and RF muscles (*p*>0.05).

### Fatiguing Exercises

**Figure 4:**
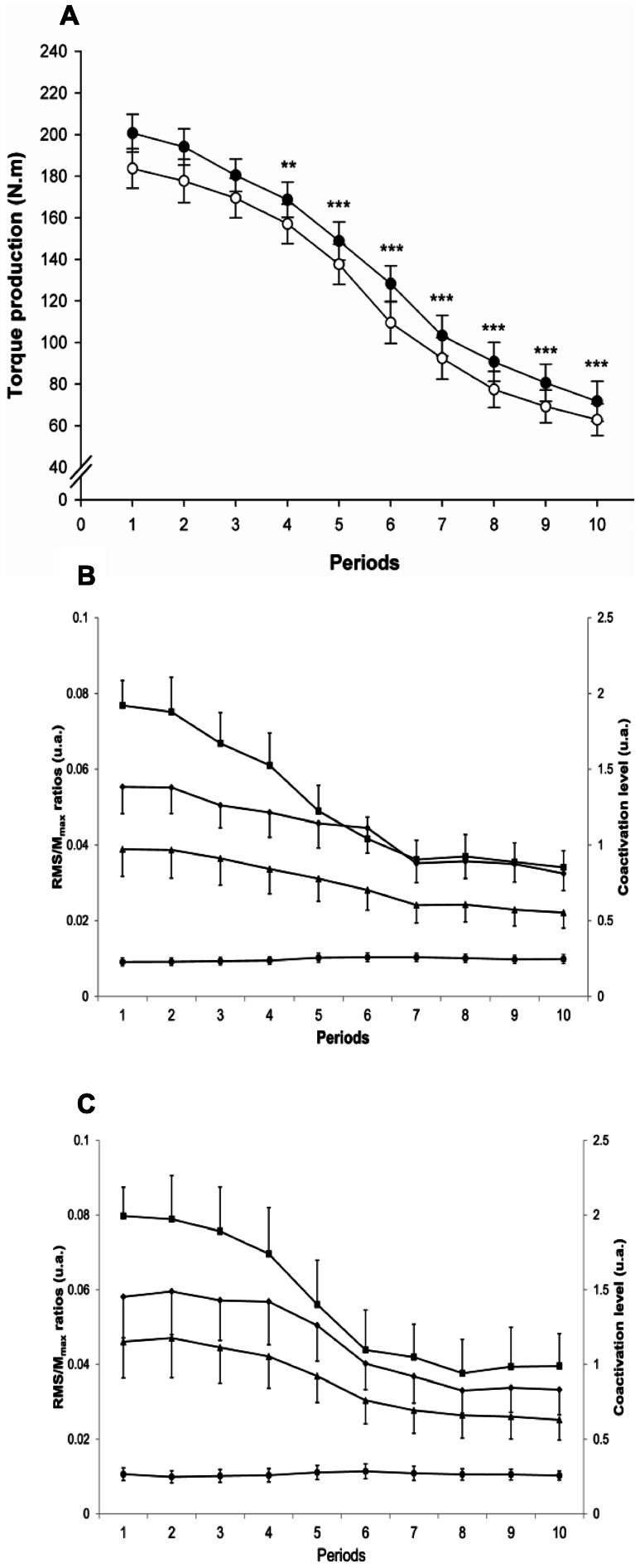
Torque production capacity and sEMG activity during the fatiguing exercises. Torque production capacity measured during the 10 periods of the first (black circles) and the second (white circles) fatiguing exercise for the exercising limb (A). Pooled data of the considered period significantly lower from the pooled data of the first period: *p*<0.01 (**) and *p*<0.001 (***). sEMG RMS/Mmax ratios of the vastus lateralis (diamonds), the vastus medialis (triangles), the rectus femoris (squares) muscles and coactivation level (circles) of the semitendinosus (ST) muscle during the 10 periods of the first (B) and second (C) fatiguing exercise. Values are mean and standard error of the mean.

No significant fatiguing exercise × periods interaction was found for the torque produced during the fatiguing exercises but significant fatiguing exercise (F = 17.27; *p*<0.001; η^2^p = 0.55) and periods (F = 87.22; *p*<0.001; η^2^p = 0.86) effects were noted. On average and over the total duration, the torque production was significantly greater during Fatigue 1 compared to Fatigue 2 (136.8±4.7 *vs.* 123.7±4.6N.m; *p*<0.001). Also, across the ten periods, the torque production for both fatiguing exercises significantly decreased comparing the first period with the fourth and so on until the last period (*p*<0.01; Figure 4 A).

Additionally, RMS/M_max_ values were found to be significantly different across the ten periods for the RF (F = 31.35; *p*<0.001; η^2^p = 0.71) between the first from the fifth and until the last period (as well as for the VM (F = 16.3; *p*<0.001; η^2^p = 0.54) and for the VL (F = 18.69; *p*<0.001; η^2^p = 0.57) between the first from the sixth and until the last period. However, no significant difference was noted for the coactivation during the fatiguing exercises (Figures 4 B and C).

## Discussion

Main findings of this study were: 1) a unilateral fatiguing exercise reduced the MVC of both the ipsilateral (EL) and the contralateral (NEL) limbs; 2) the MVC torque production decrement of both limbs appeared to be related to an overall central failure; 3) the EL also exhibited fatigue due to peripheral impairments; 4) fatigue time course differed between the EL and the NEL after a unilateral fatiguing exercise.

### Maximal Voluntary Contractions Decline in the EL and NEL

In line with previous reports in the first dorsal interosseus [8] and knee extensor muscles [7], the present work showed a decrement in maximal torque production in both the ipsilateral and contralateral limbs after a unilateral knee extensors fatiguing exercise. After the first fatiguing exercise, the MVC was significantly depressed in the EL whereas it remained unchanged in the NEL [6]. After the second fatiguing exercise, the MVC declined in both the EL and NEL demonstrating a cross-over effect of fatigue [3], [5]–[8], [11], [12]. Previous studies initially reported that a single bout of 100-second maximal effort could lead to force decline up to 25% during knee extension in the exercising limb[6], [7]. Here, we found significant maximal torque reductions in the EL of 9.6% after the first, and 17.9% after the second bout of 100-second MVC. Differences in the experimental procedures could partly explain these discrepancies since these authors have used MVCs with short rest periods (*i.e.*, 30 seconds) prior to the fatiguing exercise. Thus, it might be suggested that repetitive muscle contractions could participate to greater force reductions.

In the NEL, we observed a significant torque decline of 10.6% after the second fatiguing exercise, while a 4.9% non-significant decline occurred after the first bout. Similar non-significant force decline of ≈4.1% was also observed by Rattey et al [6]. In opposition, Martin and Rattey [7] found a significant force decline (≈13%) just after one bout of 100-second MVC exercise in the non-exercising lower limb in men while this reduction (*i.e.*, 8%) was not significant in women. In their study, participants performed MVCs of the non-exercising lower limb immediately after the fatiguing exercise of the exercising lower limb, whereas in the present study, the NEL and the EL were randomly assessed one after each other after the fatiguing exercise. Hence, it might be suggested that maximal torque of the NEL could have partially recovered when this limb was tested after the EL. This assumption is reinforced by the results of Rattey et al. [6], where no significant force reductions were found in the non-exercising lower limb when lower limbs were tested in the same way as the present study. Even though experimental procedures, as well as physical activity level of the participants and/or gender could partly account for the difference in results between our study and the previous ones [6], [7], we originally observed a difference in the time course of maximal torque decline between both limbs *(i.e.*, a progressive muscle fatigue occurring in the EL after the first fatiguing exercise and then in the NEL after the second fatiguing exercise).

### Peripheral Adaptations in the EL and NEL

Although, M-wave values of both the vastus medialis [6] and the first dorsal interosseus [8] muscles in the exercising limb have been previously reported to decrease and the values in the contralateral non-exercising limb have been reported to remain unchanged, we found here that M_max_ responses of VM, VL and RF muscles were not altered by the fatiguing exercises in both the EL and NEL [7], suggesting preservation of the neuromuscular transmission. Yet, the mechanical twitch response (Pts) evoked at rest diminished in the EL (≈24%) after the two bouts of 100-second maximal effort exercise, while it remained constant in the NEL. This result is consistent with previous reports [6], [7], although in these studies declines of over 50% were found in the exercising limb after only a single bout of 100-second MVC exercise. The use of twitch responses evoked at rest to evaluate peripheral modifications has been questioned [24] and it has been shown that potentiated responses were more sensitive for the quantification of peripheral fatigue [25], [26]. In our study, the potentiated Ptd (PtdPot) was significantly reduced in both the EL and NEL after the two bouts of 100-second MVC exercise. Even though no significant difference could be observed between limbs, a 12% decline (effect size = 0.58) of the PtdPot was noted in the EL, whereas only 2% (effect size = 0.11) was found in the NEL. Thus, our results suggest that peripheral fatigue mainly affected the EL, while this seems unlikely to occur in the NEL [6], [7]. In the absence of M_max_ impairments, this may suggest that impaired cross-bridges cycles (most probably involving Ca^2+^ handling) would have played a major role in the peripheral fatigue that occurred in the EL.

### Voluntary Activation of the EL and NEL

The reduction of voluntary activation observed in the present work is in accordance with previous literature [5]–[8]. The decline likely occurred after the second fatiguing exercise for both the EL and the NEL and was found to be correlated with the MVC loss [7]. WhileRattey et al. [6] reported, after only one bout of 100-second MVC exercise, a decline of voluntary activation of 17% in the exercising limb and 9% in the non-exercising lower limb our results showed an overall decrement of ≈10% after two bouts of 100-second maximal effort exercise. Martin and Rattey [7] reported a decline of 19 to 30% in the exercising limb and from 8 to 14% in the non-exercising limb, after one bout of 100-second MVC in women and men, respectively. In our study, the lack of significant difference in voluntary activation between the EL and the NEL is likely related to multifaceted methodological reasons. First, as argued above, these authors have used more strenuous protocols (*i.e.*, multiple MVCs with rest periods) prior to the fatiguing exercise which led to greater force declines and may also have induced greater voluntary activation decline. Second, differences can arise from the interpolation twitch technique procedures. Rattey et al. [6] used unpotentiated twitch and even though Martin and Rattey [7] quantified voluntary activation with potentiated twitch,they split their experimental design in two different days, thus maximising the potential effects of the exercise on the post fatigue measurements. In opposition with literature [6] and even though the overall activation level significantly decreased, the RMS/M_max_ ratio of the knee extensors muscles did not exhibit any change, neither in the EL nor in the NEL. This could be partly explained by the fact that assessment of voluntary activation by means of twitch interpolation technique is more reproducible than RMS/M_max_ ratios [26], [27]–[29]. Moreover, one limitation of this study relates to the time interval between the end of the sustained contraction and both the voluntary activation and MVC assessments. Indeed, and in agreement with previous reports on knee extensors [7] and elbow flexor muscles [30], [31], [32], we observed a loss of torque production respectively of 64.2% and 65.1% at the end of each bout of 100-second MVC exercise. However, the MVC torque decrements in the EL were −9.63% at MID and −9.24% at POST. Although these observations demonstrate that the participants had no pacing strategy when performing the first and second fatiguing exercises [20], a partial recovery could have occurred, thus explaining the non significant change of the RMS/M_max_ ratios. Finally, not only the electrical activity of agonist muscles was not altered, but the coactivation level of the ST muscle remained unchanged. Although this observation is unique in the literature regarding the cross-over effect of fatigue, we only recorded the sEMG of the ST muscle amongst the hamstrings muscles Interestingly, the present results confirmed that, since no peripheral fatigue occurred in the NEL, the decline of MVC in the NEL relied on a cross-over effect of fatigue likely occurring at a central level whereas the MVC impairment in the EL might likely relate to both central and peripheral fatigue factors.

### Possible Mechanisms Explaining Central Failure and Limbs Interactions

Central failure encompasses adjustments at both spinal and supraspinal levels that induce reduced excitation of the motorneurone pool or decrease motorneurones responsiveness [33]. Although the twitch interpolation technique does not permit differentiation of the supraspinal from spinal mechanisms responsible for central fatigue [28], spinal inhibition arising from muscles afferents (muscle spindles and group III and IV afferents) in the EL could disturb the motorneurones excitation of the contralateral NEL. Indeed, it has been shown that unilateral task can depress H-reflex of the contralateral homologous muscle in both the upper [34], [35] and lower limbs [36]. Then, the reduced MVC of the NEL observed in our study could result from altered activity at spinal level of the ipsilateral EL through commissural interneurons.

Supraspinal fatigue has been related to an inadequate cortical output [30] and group III and IV muscles afferents are likely to limit the circuits that generate voluntary drive [37], [38]. In addition, there is recent evidence that intracortical inhibition increased during a 2-minute MVC of the elbow flexor muscles [31]. In this context, the MVC decline of the NEL observed in the present study could also be related to inter-hemispheric neural regulation through transcallosal pathways [39]. Nonetheless, there is evidence that supraspinal fatigue was minimal in the elbow flexor muscles [5] and spinal mechanisms were proposed as the major contributor of central fatigue during a 2-minute MVC of the elbow flexor muscles [5]. Based on these observations and recent conclusions [40], we assume that changes at a spinal cord are likely to contribute to the overall voluntary activation decline. Although speculative, this regulation from spinal crossed reflex pathways during fatiguing exercise [11] has been observed after unilateral strength training [41] and requires further investigation. Our results are nevertheless of importance for functional activities suggesting that the cross-over effect of fatigue would occur to balance bilateral activity for two-limb coordinated and automatic tasks (*e.g.*, balance, locomotion…) and more generally to maintain lower limbs homeostasis. Recent studies have highlighted that unilateral fatigue of lower limb musculature disturbs bipedal postural control [10], [11], [42], [43]. In addition, unilateral fatigue induced by single-leg squats has been shown to provoke a cross-over effect of central fatigue to the contralateral limb during single-leg landings in elite female athletes which is substantially sufficient to support dangerous postural adjustments [9]. In this instance, we suggest that the central nervous system develops a compensating strategy to reduce MVC of the NEL through lower voluntary activation to cope with the weaker MVC of the EL, and therefore to warrant bilateral coordination of the lower limbs. The current data may have also direct implications in both the therapeutic and the physical training contexts where repetitive unilateral exercise is often performed for strengthening purpose. For example, neural adaptations are likely to be an explanative mechanism of the cross-education effect observed when a unilateral strength training leads to a strength gain to the non-trained contralateral limb [44]–[46]. Here, we emphasised that cross-over effects of fatigue induced by a unilateral exercise was of central origin. Considering that muscle fatigue contributes at least in part to the strength training stimulus [47], it might be speculated that unilateral exercise would activate central pathways that facilitate cross-transfer in the contralateral limb. As a consequence, contrary to some previous beliefs [3]–[5], [8] and in agreement with others [6], [7], [9] we concluded that the magnitude of the cross-over effect of central fatigue is important in the lower limbs.

### Conclusion

This study has highlighted that a unilateral fatiguing exercise consisting of two bouts of 100-second MVC knee extension leads to cross-over fatigue to the contralateral limb and that the time course of muscle fatigue differed between both limbs. It seems that peripheral fatigue of the EL arose through adaptations involving intramuscular processes located beyond the sarcolemma whereas spinal crossed reflex pathways were proposed as the main factor of voluntary activation failure of the EL and the NEL. However, identification of central mechanism that is responsible for the interlimbs adjustment seem worthy of consideration and exploration.
